# Induction of apoptosis through extrinsic/intrinsic pathways and suppression of ERK/NF‐κB signalling participate in anti‐glioblastoma of imipramine

**DOI:** 10.1111/jcmm.15022

**Published:** 2020-03-09

**Authors:** Fei-Ting Hsu, I‐Tsang Chiang, Wei‐Shu Wang

**Affiliations:** ^1^ Department of Biological Science and Technology China Medical University Taichung Taiwan; ^2^ Department of Radiation Oncology Show Chwan Memorial Hospital Changhua Taiwan; ^3^ Department of Radiation Oncology Chang Bing Show Chwan Memorial Hospital Lukang Taiwan; ^4^ Department of Medical Imaging and Radiological Sciences Central Taiwan University of Science and Technology Taichung Taiwan; ^5^ Department of Medicine National Yang‐Ming University Hospital Yilan Taiwan; ^6^ School of Medicine National Yang‐Ming University Taipei Taiwan

**Keywords:** ERK, extrinsic/intrinsic apoptosis, glioblastoma, imipramine, NF‐κB

## Abstract

Glioblastomas are the most aggressive type of brain tumour, with poor prognosis even after standard treatment such as surgical resection, temozolomide and radiation therapy. The overexpression of the nuclear factor kappa‐light‐chain‐enhancer of activated B cells (NF‐κB) in glioblastomas is recognized as an important treatment target. Thus, an urgent need regarding glioblastomas is the development of a new, suitable agent that may show potential for the inhibition of extracellular signal‐regulated kinase (ERK)/NF‐κB–mediated glioblastoma progression. Imipramine, a tricyclic antidepressant, has anti‐inflammatory actions against inflamed glial cells; additionally, imipramine can induce glioblastoma toxicity via the activation of autophagy. However, whether imipramine can suppress glioblastoma progression via the induction of apoptosis and blockage of ERK/NF‐κB signalling remains unclear. The main purpose of this study was to investigate the effects of imipramine on apoptotic signalling and ERK/NF‐κB–mediated glioblastoma progression by using cell proliferation (3‐(4,5‐Dimethylthiazol‐2‐yl)‐2,5‐diphenyltetrazolium bromide [MTT] assay), flow cytometry, Western blotting, and cell invasion/migration assay analysis in vitro. The ERK and NF‐κB inhibitory capacity of imipramine is detected by NF‐κB reporter gene assay and Western blotting. Additionally, a glioblastoma‐bearing animal model was used to validate the therapeutic efficacy and general toxicity of imipramine. Our results demonstrated that imipramine successfully triggered apoptosis through extrinsic/intrinsic pathways and suppressed the invasion/migration ability of glioblastoma cells. Furthermore, imipramine effectively suppressed glioblastoma progression in vivo via the inhibition of the ERK/NF‐κB pathway. In summary, imipramine is a potential anti‐glioblastoma drug which induces apoptosis and has the capacity to inhibit ERK/NF‐κB signalling.

## 1 INTRODUCTION

Glioblastomas, also known as grade IV gliomas, are the most common and aggressive type of tumour of the central nervous system (CNS) in adults.^1^ Rapid tumour progression, as an unfavourable prognostic factor, is related to poor outcomes in patients with glioblastomas.^2^, ^3^ Genetic and epigenetic alterations modulate the hyperactivation of oncogenic signalling transduction and are associated with the pathogenesis of glioblastomas. The upstream oncogenic kinases trigger downstream transcription factor‐regulated oncogene expression, promoting the progression of glioblastomas.^1^, ^4^, ^5^ The blockage of oncogenic signalling transduction contributes to the inhibition of tumour progression.

Apoptosis is programmed cell death which triggers cell death by extrinsic and intrinsic (mitochondrial) apoptotic pathways.^6^ Chemotherapeutic agents modulate the reduction in tumour progression through the induction of apoptosis by deoxyribonucleic acid (DNA) damage. The high expressions of DNA repair and anti‐apoptotic proteins diminish the anticancer efficacy of chemotherapy through the repair of DNA lesions and blockage of apoptotic signalling transduction.^7^, ^8^ High expressions of DNA repair and anti‐apoptotic proteins were associated with poor survival in patients with glioblastoma.^9^, ^10^

The nuclear factor kappa‐light‐chain‐enhancer of activated B cell (NF‐κB) p50/p65 heterodimer is the oncogenic transcription factor driving the expression of oncogenes and plays the critical mediator role in tumour progression. Constitutive NF‐κB activation controls the expression of tumour progression‐associated proteins which participate in tumour cell growth, anti‐apoptosis, angiogenesis and metastasis.^11^, ^12^, ^13^ In addition, the expression of several DNA repair proteins is also linked to NF‐κB activation.^14^, ^15^ Puliyappadamba et al demonstrated that glioblastoma had higher NF‐κB activation compared to normal brain and lower grade glioma.^1^

NF‐κB activation cannot be inhibited with chemotherapy or radiotherapy.^16^, ^17^ Therefore, the development of complementary agents which repress NF‐κB signalling may offer therapeutic benefits for patients with glioblastomas. Anticancer effects and antidepressant mechanisms for glioma have been demonstrated.^18^, ^19^ Fluoxetine, the antidepressant of the selective serotonin reuptake inhibitor (SSRI), was demonstrated to reduce NF‐κB activation and sensitize glioblastomas to the chemotherapeutic agent temozolomide (TMZ).^20^ Fluoxetine also induced apoptosis through the calcium‐mediated intrinsic apoptotic pathway in glioblastoma.^19^

Long‐term use of tricyclic antidepressants (TCAs) has been indicated to decrease glioma risk.^21^ Imipramine, the TCA, has been shown to induce an accumulation of reactive oxygen species and inhibits NF‐κB p65 gene expression in glioblastoma.^22^ Jeon et al^23^ presented imipramine‐inhibited tumour cell growth through autophagy in glioblastoma in vitro. Furthermore, to overcome glioblastoma, whether imipramine can cross blood‐brain barrier (BBB) is the major issue of drug selection. As compared to various antidepressant drug, such as duloxetine, fluoxetine and mirtazapine, the BBB penetration ability of imipramine was relatively better.^24^ However, whether the induction of apoptosis and suppression of NF‐κB signalling are associated with the imipramine‐inhibited progression of glioblastomas is ambiguous. The major purpose of this study was to verify the efficacy and mechanism of imipramine on tumour progression in glioblastoma in vitro and in vivo.

## 2 MATERIALS AND METHODS

### 2.1 Chemicals and reagents

Imipramine, 3‐(4,5‐Dimethylthiazol‐2‐yl)‐2,5‐diphenyltetrazolium bromide (MTT) and dimethyl sulphoxide (DMSO) were purchased from Sigma‐Aldrich. The concentration of imipramine stock solution was prepared as 100 mmol/L and stored at −20°C. Minimum essential medium Eagle (MEM), foetal bovine serum (FBS), l‐glutamine, sodium pyruvate and penicillin‐streptomycin were all bought from Gibco/Life Technologies.^25^ Hygromycin and jetPEI™‐DNA transfection reagent were purchased from Santa Cruz Biotechnology and Polyplus Transfection, respectively. d‐luciferin and isoflurane were obtained from Promega and GM‐GLOBAL, respectively. Propidium iodide and RNase were purchased from Biovision and Thermo Fisher Scientific, respectively.

### 2.2 Cell culture

Human U‐87 MG and GBM8401 cells used in this study were provided by Professor Ruei‐Ming Chen, Taipei Medical University, Taiwan and Professor Jing‐Gung Chung, China Medical University, Taiwan, respectively. Cells were maintained in MEM medium or RPMI‐1640 medium with 10% FBS, 100 IU/mL penicillin, 100 mg/mL streptomycin and 1 mmol/L sodium pyruvate, and then incubated at 37°C in a humidified atmosphere of 5% CO_2_.^26^ Cells were grown to 70%‐80% confluence before drug treatment.

### 2.3 Cell viability assay and morphological changes

U‐87 MG and GBM8401 cells were seeded on 96‐well plate at 2 × 10^4^ cells/well for 24 hours, followed by imipramine treatment for another 24 or 48 hours. MTT reagent was dissolved in DMSO at 5 mg/mL concentration. Next, the medium was refreshed by MTT solution (1:9 = MTT reagent: MEM medium) and incubated for 4 hours. The supernatants were then removed carefully, and formazan crystals were dissolved by DMSO. Finally, the absorbance was measured by Multiskan™ GO Microplate Spectrophotometer (Thermo Fisher Scientific) at 570 nm.^27^ A phase‐contrast microscope was used to determine morphological shrinkage after 40 or 80 µmol/L imipramine treatments for 48 hours.

### 2.4 Plasmid transfection of U‐87 MG cells

The vector containing NF‐κB‐luciferrase 2 vector (pNF‐κB/*luc2*) was prepared in advance (Promega). U‐87 MG cells were grown on a 6 cm plate and maintained at 70% confluency before the transfection process. Cationic polymer transfection reagent (Polyplus transfection) was used to transport these NF‐κB/luc2 plasmids into the intrathecal region. First, 10 µL jetPEI™ reagent in 250 µL of NaCl buffer was added into plasmid solution (5 µg plasmids with 250 µL of NaCl buffer) followed by incubation for 25 minutes at room temperature. Second, the mixture was added onto a 6‐cm plate and incubated for one day. Third, cells were then selected by hygromycin B 200 µg/mL for 2 weeks as a U87/*NF‐κB*/*luc2* stable clone for further investigation.^28^, ^29^, ^30^

### 2.5 Sub‐G1 phase (apoptosis) assays

Briefly, U‐87 MG and GBM8401 cells were placed at a concentration of 5 × 10^5^ cells/well in 6‐well plates overnight, and cells were incubated with imipramine (0, 40 and 80 µmol/L) for another 48 hours and were harvested, washed with phosphate‐buffered saline and fixed in 70% ethanol overnight at −20°C. After fixation, cells were then re‐suspended in solution containing 40 µg/mL PI, 100 µg/mL RNase A and 1% Triton X‐100 and incubated at 37°C for 30 minutes. After staining, cells were measured by flow cytometry (FACS) (BD Biosciences, FACS Calibur) and analysed with FlowJo software (version 7.6.1; FlowJo LLC).^29^, ^31^

### 2.6 Annexin V/PI apoptosis analysis

Briefly, U‐87 MG and GBM8401 cells were placed at a concentration of 5 × 10^5^ cells/well in 6‐well plates overnight, and cells were incubated with imipramine (0, 40 and 80 µmol/L) for another 48 hours. Cells were then washed, harvested and stained by an Annexin VFITC apoptosis detection kit (Vazyme Biotech Co. Ltd). After staining, cells were measured by flow cytometry and analysed with FlowJo software.^29^, ^31^

### 2.7 Measurements of caspase‐3 and caspase‐8 activities

U‐87 MG and GBM8401 cells were placed at a concentration of 5 × 10^5^ cells/well in 6‐well plates overnight, and cells were incubated with imipramine (0, 40 and 80 µmol/L) for another 48 hours. Cells were collected, washed with PBS and re‐suspended in 1 µL of substrate solution containing CaspGlow Fluorescein active Caspase‐3 (BioVision) for caspase‐3 activity measurement or containing CaspGlow fluorescein active caspase‐8 for caspase‐8 activity measurement before being incubated at 37°C for 30 minutes. Cells from each treatment were washed, and caspase‐3 and ‐8 activities were analysed by flow cytometry as described previously.^31^

### 2.8 Measurements of Fas and Fas‐L activities

U‐87 MG and GBM8401 cells were placed at a concentration of 5 × 10^5^ cells/well in 6‐well plates overnight, and cells were incubated with imipramine (0, 40 and 80 µmol/L) for another 48 hours. Cells were collected, washed with PBS and re‐suspended in 1 µL of substrate solution containing Anti‐Fas‐FITC (Thermo Fisher Scientific) for Fas activity measurement or containing anti–Fas‐L‐PE for Fas‐L activity measurement before being incubated at 37°C for 30 minutes. Fas and Fas‐L activities were analysed by flow cytometry as described previously.^31^

### 2.9 Measurements of ROS, intracellular Ca^2+^ and mitochondrial membrane potential (ΔΨ_m_)

U‐87 MG and GBM8401 cells were placed at a concentration of 5 × 10^5^ cells/well in 6‐well plates overnight, and cells were incubated with imipramine (0, 40 and 80 µmol/L) for another 48 hours. Cells were isolated and re‐suspended with 500 µL of dichlorodihydrofluorescein diacetate (DCFH‐DA; 10 µmol/L) and kept in the dark for 60 minutes, and were then analysed for reactive oxygen species (ROS) production.^31^, ^32^ For intracellular Ca^2+^ concentration measurement, cells were isolated and re‐suspended with 500 µL of Fluo‐3/AM (2.5 µg/mL) and maintained in the dark for 30 minutes for intracellular Ca^2+^ concentrations. For ΔΨ_m_, cells were isolated and re‐suspended with 500 µL of DiOC_6_ (4 µmol/L), maintained in the dark for 30 minutes and were analysed for the levels of ΔΨ_m_.^29^ Total viable cells with ROS, Ca^2+^ and ΔΨ_m_ were measured by flow cytometry as previously described.^33^

### 2.10 In vitro and in vivo NF‐κB reporter assay

Briefly, U87/*NF‐κB/luc2* cells were seeded in 96‐well plates with 2 × 10^4^ cells/well overnight. Cells were then incubated with imipramine (0, 40, and 80 µmol/L) for 48 hours. Before the bioluminescent imaging scan (BLI), 96‐well mediums were replaced and incubated by 100 µL d‐luciferin (500 µmol/L) for 5 minutes. NF‐κB signal from cells was collected by IVIS200 Imaging System (Xenogen) for 1 minute and quantified into photons per second using Living Image software (Xenogen). The activation of NF‐κB was normalized by viability results from MTT assay.^27^ Animal NF‐κB activation signals were also acquired by IVIS after 100 µL d‐luciferin (150 mg/kg) injection as previously described.^34^

### 2.11 Invasion and migration assay

The assessment of in vitro invasion and migration activities were carried out using matrigel‐coated or uncoated transwell cell culture chambers (8 μm pore size) as described previously.^35^, ^36^ Briefly, U‐87 MG and GBM8401 cells (3 × 10^6^ cells/well) in serum‐free medium were maintained in a 10‐cm dish and were incubated with imipramine (0, 40 and 80 µmol/L) for 48 hours. For the migration assay, cells were placed on the top of the well with a membrane. The cells on the upper surface of the membrane were removed by a cotton swab, and invaded cells on the lower surface were fixed with 4% cold formaldehyde, stained with 0.1% crystal violet and then photographed. For the invasion assay, the same assay was used, except that the membrane was coated with matrigel as described previously. The migrated and invaded cells in the chamber were counted under a light microscope (Nikon ECLIPSE Ti‐U) at a magnification of ×100.

### 2.12 Wound healing assay

U‐87 MG and GBM8401 cells (5 × 10^5^ cells/well) maintained in 6‐well plates were grown to complete confluency. Cell monolayers were scraped using a sterile yellow micropipette tip and washed with PBS three times. Cells were then cultured in DMEM medium containing 0, 40 or 80 µmol/L of imipramine for 48 hours. Cells were examined and photographed using an inverted microscope (Nikon ECLIPSE Ti‐U) as described previously.^37^

### 2.13 Western blotting analysis

U‐87 MG and GBM8401 cells were placed at a concentration of 3 × 10^6^ cells/well in 10‐cm plates overnight, and cells were incubated with imipramine (0, 40 and 80 µmol/L) for another 48 hours. Proteins were lysed by NP‐40 lysis buffer, separated by 10%‐12% SDS‐page and transferred to polyvinylidene difluoride (PVDF) membranes (EMD Millipore) as previously described.^35^, ^38^ The protein level changes of O‐6‐Methylguanine‐DNA Methyltransferase (MGMT) (Thermo Fisher Scientific), Phospho‐p44/42 MAPK (Erk1/2) (Cell signaling, Danvers), T‐ERK (Santa Cruz), Matrix metallopeptidase‐2 (MMP‐2) (ProteinTech Group Inc), MMP‐9 (EMD Millipore), uPA (Abcam plc.), vascular endothelial growth factor (VEGF) (EMD Millipore), Cyclin D1 (Thermo Fisher Scientific), X‐linked inhibitor of apoptosis protein (XIAP) (Thermo Fisher Scientific) and β‐actin (Thermo Fisher Scientific) after imipramine treatment were determined.

### 2.14 Animal

Twenty male athymic BALB/c nu/nu mice at 6‐8 weeks of age were obtained from the National Laboratory Animal Center (Taipei, Taiwan). The animals were maintained in standard vinyl cages in a filtered laminar airflow room at room temperature and fed with autoclaved water and food. The Animal Care and Use Committee in Taipei Medical University approved the study (ID: LAC2018‐0181). U87/*NF‐*κ*B/luc2* cells (1 × 10^7^ cells/mice) were re‐suspended in 150 μL mixture (serum‐free DMEM:matrigel = 2:1), and then, the cell suspension was implanted subcutaneously into the right leg of nude mice. Tumour size of each animal was measured using callipers every 3 days. Tumour volume was calculated by the equation: *V* = *L* × *W* ^2^ × 0.523 (where *V* is the volume, *L* is the length, and *W* is the width).^33^ When tumour volume in each animal reached 100 mm^3^, experimental treatments were started. Animals were randomly divided into two groups (5 animals in each group). Mice were fed with either vehicle (0.1% DMSO by gavage) only or imipramine (10 mg/kg by gavage) every day until the 21st day (after initiation of therapy). The experimental protocol was summarized in Figure 7A. Tumours from each mouse were removed, photographed, measured and weighed after being killed on day 21.

### 2.15 Haematoxylin and eosin stain

Liver tissues were fixed in 10% formalin and embedded in paraffin. Paraffin‐embedded specimens were then sliced, deparaffinized, rehydrated and stained with haematoxylin and eosin (H&E) by Bio‐Check Laboratories Ltd as previously described.^39^

### 2.16 Immunohistochemistry staining

The immunohistochemistry (IHC) assay was conducted on mice tumour tissue to detect and evaluate MMP‐2, MMP‐9, uPA, VEGF, XIAP, Cyclin D1, P‐ERK, p65‐pNF‐κB and MGMT expression using the methods described previously.^34^ Anti–NF‐κB‐p65 (Phospho‐Ser276) was obtained from Signalway Antibody.

### 2.17 Statistical analysis

Data were expressed as mean ± SEM. Student's *t* test was used to compare the means of vehicle and imipramine groups by Excel 2017 (Microsoft). A *P* value of <.05 was considered statistically significant.^40^

## 3 RESULTS

### 3.1 Imipramine decreased cell viability and induced cell morphological changes of U‐87 MG and GBM8401 glioblastoma cells

**Figure 1:**
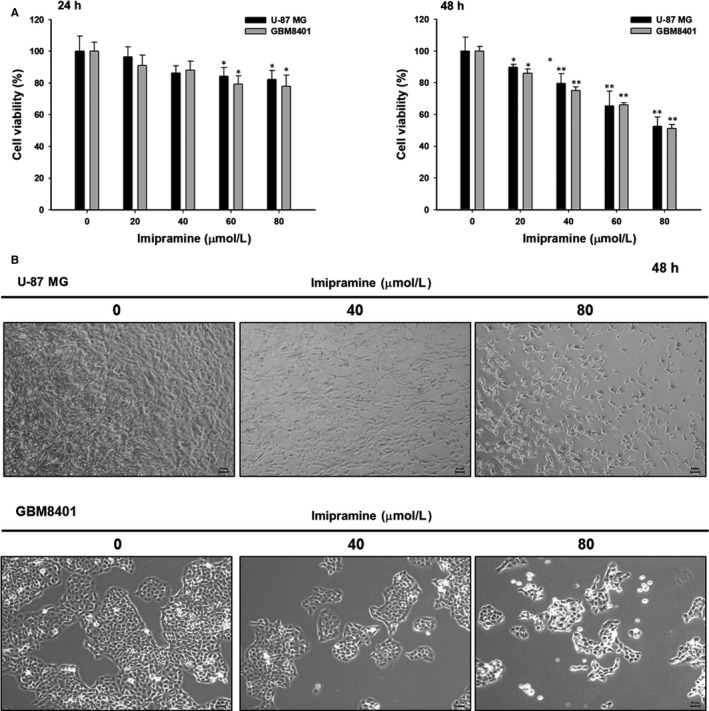
Cell viability was decreased, and cell morphology was changed by imipramine in glioblastoma cells. Cells were treated with imipramine concentrations of 0, 20, 40, 60 or 80 µmol/L for 24 or 48 h. A, Cell viability was assayed by 3‐(4,5‐dimethylthiazol‐2‐yl)‐2,5‐diphenyltetrazolium bromide (MTT) assay. B, Cell morphology was examined by contrast‐phase microscopy. Significant differences from the control and imipramine‐treated groups were recorded at *P < .05 and **P < .01

U‐87 MG and GBM8401 glioblastoma cells were treated with various concentrations of imipramine for 24 or 48 hours; then, the total viable cells were determined and the cell morphology examined. Figure 1A indicated that imipramine decreased the percentage of viable cells, and these effects were concentration‐dependent in two glioblastoma cells. As compared to 24 hours, the cytotoxicity effect of imipramine is more obvious after 48‐hour treatment. Thus, we performed 48‐hour treatment of imipramine in further experiment. In addition, 40 and 80 µmol/L of imipramine markedly induced cell morphological changes based on cell shrinkage and debris appearance on both U‐87 MG and GBM8401 glioblastoma cells (Figure 1B).

### 3.2 Imipramine decreased the cell mobility, migration and invasion ability of U‐87 MG and GBM8401 glioblastoma cells

**Figure 2:**
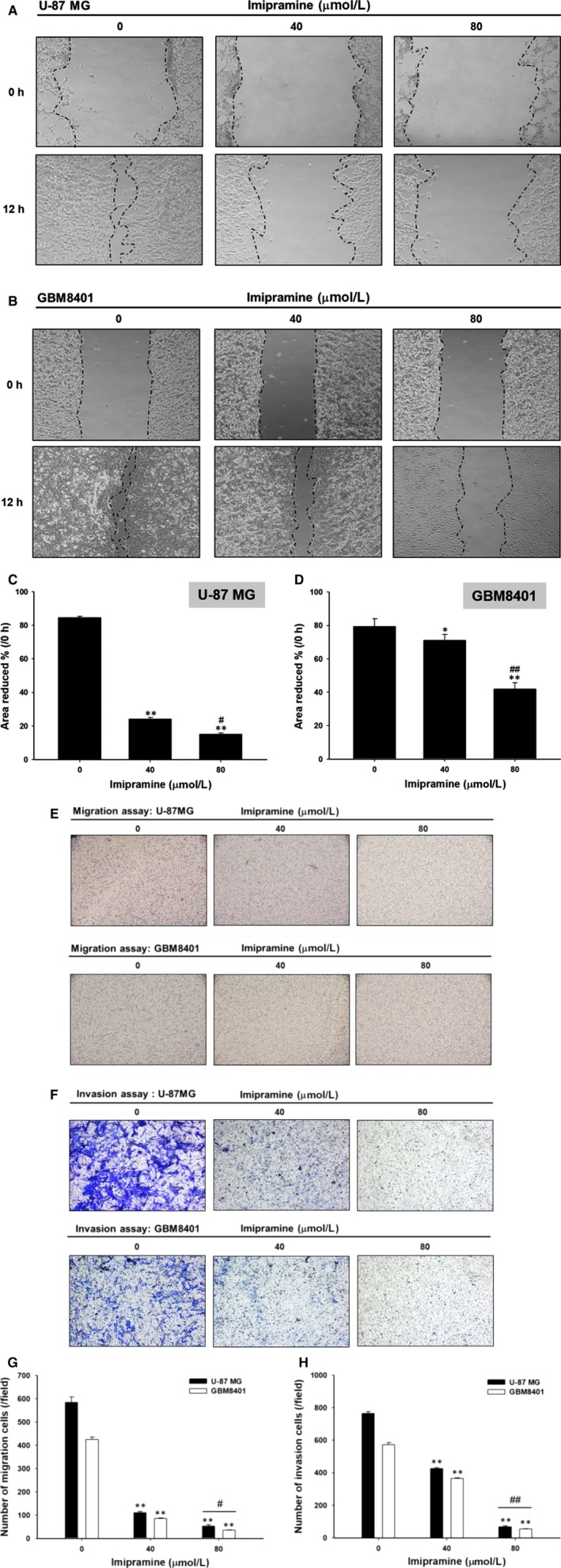
Cell mobility, migration and invasion ability of glioblastoma cells were suppressed by imipramine. U‐87 MG and GBM8401 were grown in 6‐well plates until 80% confluence, and cell monolayers were scraped and incubated in medium containing 0, 40 and 80 µmol/L of imipramine for 48 h. After incubation, the closure of the gap generated by the scraping was assayed as described in the Section 2. A, B, The representative figures for the closures of scraped areas. C, D, The percentage of area reduction was calculated by Image J. Transwell assays were performed to detect the (E) migration and (F) invasion activity. The quantification of (G) migrated and (H) invaded cells was assayed by Image J. *P < .05 and **P < .01: significant differences between imipramine‐treated groups and the control as analysed by Student's *t* test. ^#^ *P* < .05 and ^##^ *P* < .01: significant differences between 40 µmol/L imipramine‐treated groups and 80 µmol/L imipramine‐treated groups

In Figure 2A,B, the results show that the closure of the scraped area in the imipramine‐treated cells was slower than that of the control at both treatment doses in both types of glioblastoma cells. The quantification results indicated the area without cells was markedly decreased in controls as compared to imipramine treatment (Figure 2C,D). The measurement of cell migration and invasion was performed by using transwell cell migration and invasion assays. Imipramine significantly (*P* < .05) inhibited cell migration and invasion in a dose‐dependent manner in both types of glioblastoma cells (Figure 2E,F). The quantification results indicated that imipramine inhibited cell migration by 70%‐80% and cell invasion by 30%‐50% at 48 hours compared to control cells (Figure 2G,H).

### 3.3 Imipramine‐induced apoptotic cell death in U‐87 MG and GBM8401 glioblastoma cells

**Figure 3:**
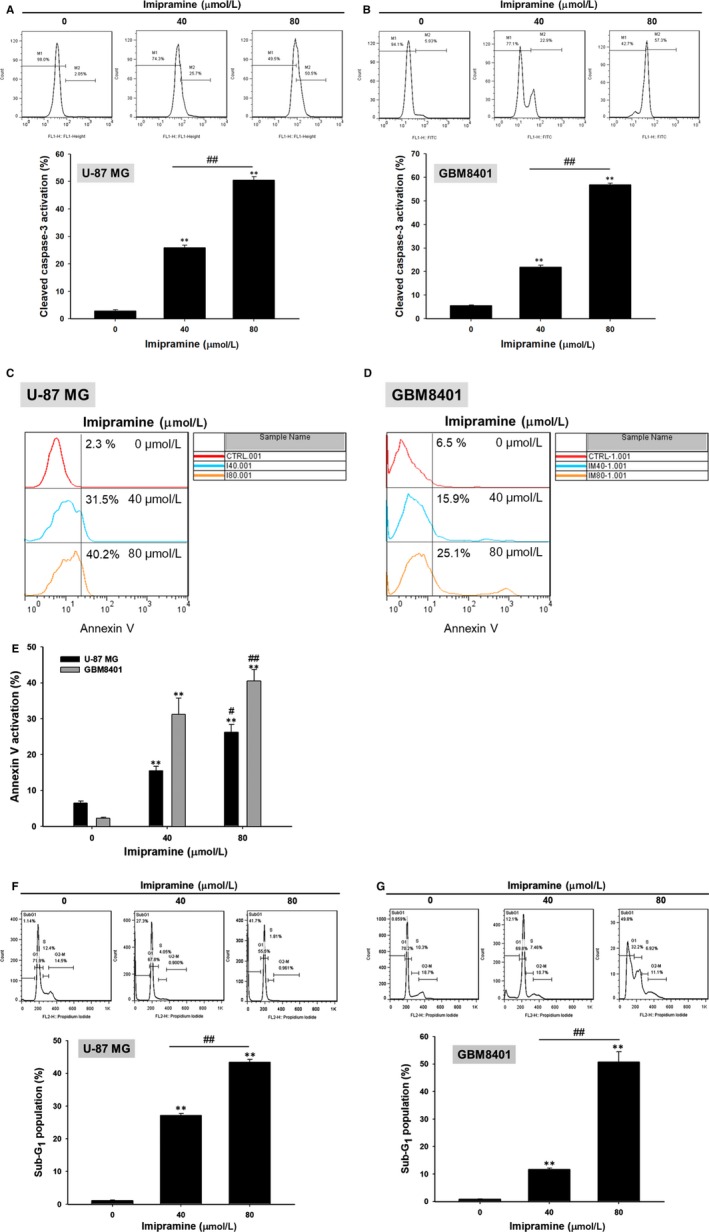
Cleaved caspase‐3 activation, annexin V activation and sub‐G1 accumulation of glioblastoma cells induced by imipramine. U‐87 MG and GBM8401 were grown in 6‐well plates until 80% confluence. After 0, 40 and 80 µmol/L imipramine treatment for 48 h, (A, B) cleaved caspase‐3 activation, (C‐E) annexin V and (F, G) sub‐G1 accumulation on U‐87 MG and GBM8401 were measured by flow cytometry. **P < .01: significant difference between imipramine‐treated groups and the control as analysed by Student's *t* test. ^##^ *P* < .01: significant difference between 40 µmol/L imipramine‐treated groups and 80 µmol/L imipramine‐treated groups

We further investigated whether imipramine may trigger an apoptosis effect of U‐87 MG and GBM8401 glioblastoma cells. As shown in Figure 3A,B, the apoptosis marker cleaved caspase‐3 was noticeably activated by a dose elevation of imipramine. In annexin V staining, apoptosis effect was both induced in U‐87 MG and GBM8401 cells by imipramine after 48‐hour treatment (Figure 3C‐E). In addition, the sub‐G1 population, which was recognized as apoptotic cell death, was also increased by imipramine (Figure 3F,G). These results proved that an apoptosis effect was effectively triggered by imipramine on both types of glioblastoma cells.

### 3.4 Imipramine triggered the death receptor‐dependent extrinsic apoptosis effect of U‐87 MG and GBM8401 glioblastoma cells

**Figure 4:**
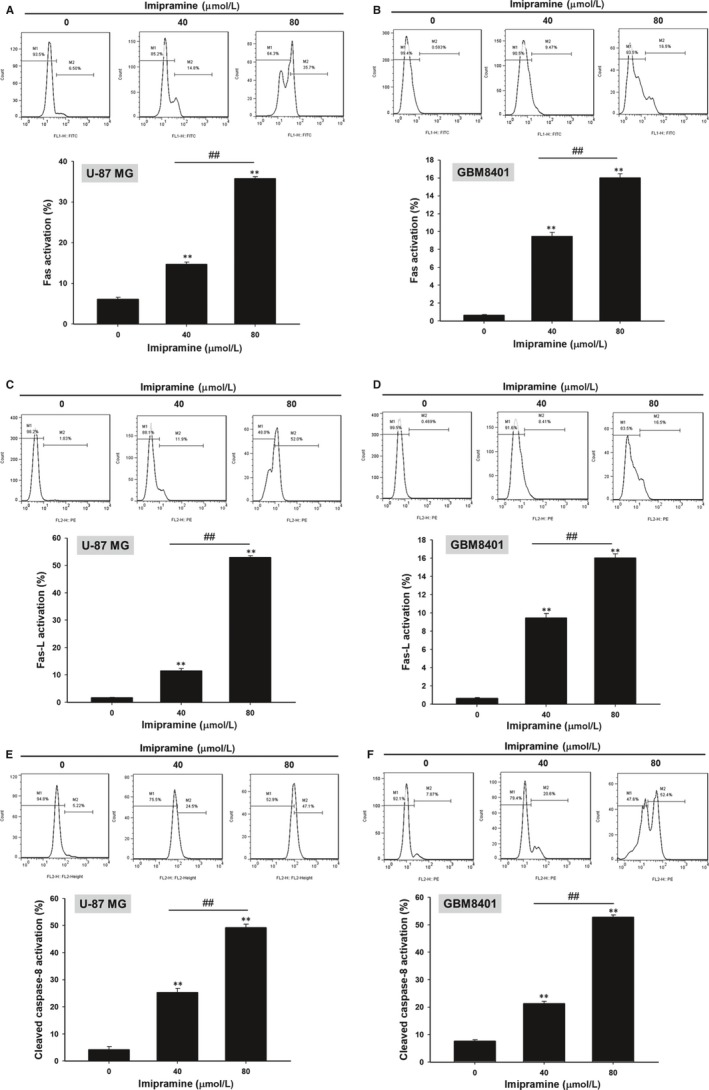
Fas activation, Fas‐L activation and cleaved caspase‐8 activation of glioblastoma cells were increased by imipramine. U‐87 MG and GBM8401 were grown in 6‐well plates until 80% confluence. After 0, 40 and 80 µmol/L imipramine treatment for 48 h, (A, B) Fas activation, (C, D) Fas‐L activation and (E, F) cleaved caspase‐8 activation were evaluated by flow cytometry. **P < .01: significant difference between imipramine‐treated groups and the control as analysed by Student's *t* test. ^##^ *P* < .01: significant difference between 40 µmol/L imipramine‐treated groups and 80 µmol/L imipramine‐treated groups

To identify whether the apoptosis effect of imipramine was modulated by extrinsic death receptor‐dependent apoptosis signalling, we investigated several extrinsic apoptosis markers. First of all, Fas activation was increased by imipramine on U‐87 MG and GBM8401 cells (Figure 4A,B). The Fas corresponded ligand Fas‐L was also markedly activated by imipramine, as shown in Figure 4C,D in both types of glioblastoma cells. Moreover, we found that the downstream molecular of Fas and Fas‐L, cleaved caspase‐8, was also increased by imipramine on two types of glioblastoma cells (Figure 4E,F).

### 3.5 Imipramine‐induced mitochondrial‐dependent intrinsic apoptosis effect of U‐87 MG and GBM8401 glioblastoma cells

**Figure 5:**
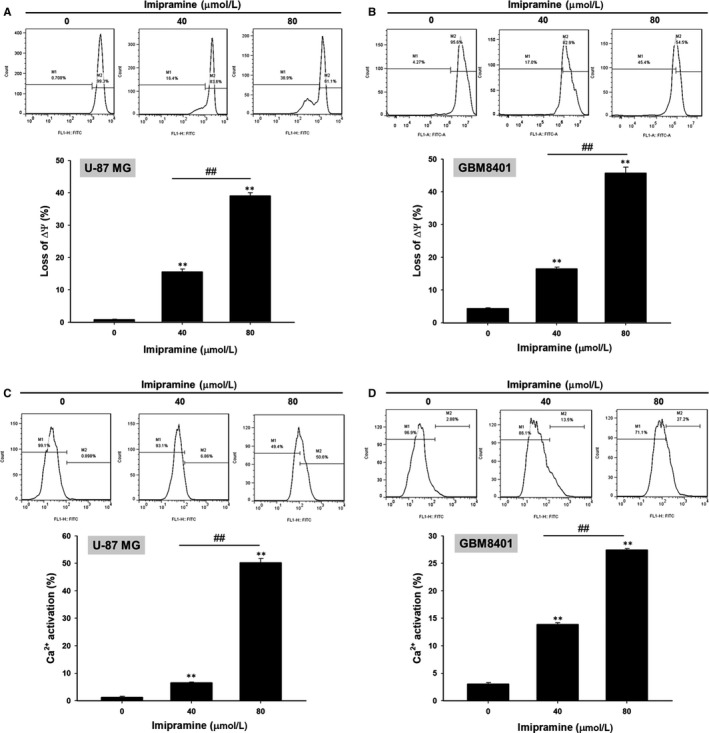
Losses of ΔΨ_m_ and concentration of intracellular Ca^2+^ of glioblastoma cells were increased by imipramine. U‐87 MG and GBM8401 were grown in 6‐well plates until 80% confluence. After 0, 40 and 80 µmol/L imipramine treatment for 48 h, (A, B) the losses of ΔΨ_m_ and (C, D) Ca^2+^ concentration were evaluated by flow cytometry. **<0.01: significant difference between imipramine‐treated groups and the control as analysed by Student's *t* test. ^##^ *P* < .01: significant difference between 40 µmol/L imipramine‐treated groups and 80 µmol/L imipramine‐treated groups

To further validate whether imipramine‐induced apoptosis is mediated by mitochondria‐dependent intrinsic apoptosis, we performed MMP (ΔΨ_m_) and Ca^2+^ concentration analysis. As shown in Figure 5A,B, the loss of ΔΨ_m_ was increased 15%‐45% in imipramine‐treated cells as compared to controls. Results from Figure 5C,D indicate that Ca^2+^ production increased in imipramine treatments, which may indicate that imipramine‐induced cell apoptosis is associated with Ca^2+^ production.

### 3.6 Imipramine inhibited tumour progression via targeting ERK/NF‐κB and its associated protein levels in U‐87 MG and GBM8401 glioblastoma cells

**Figure 6:**
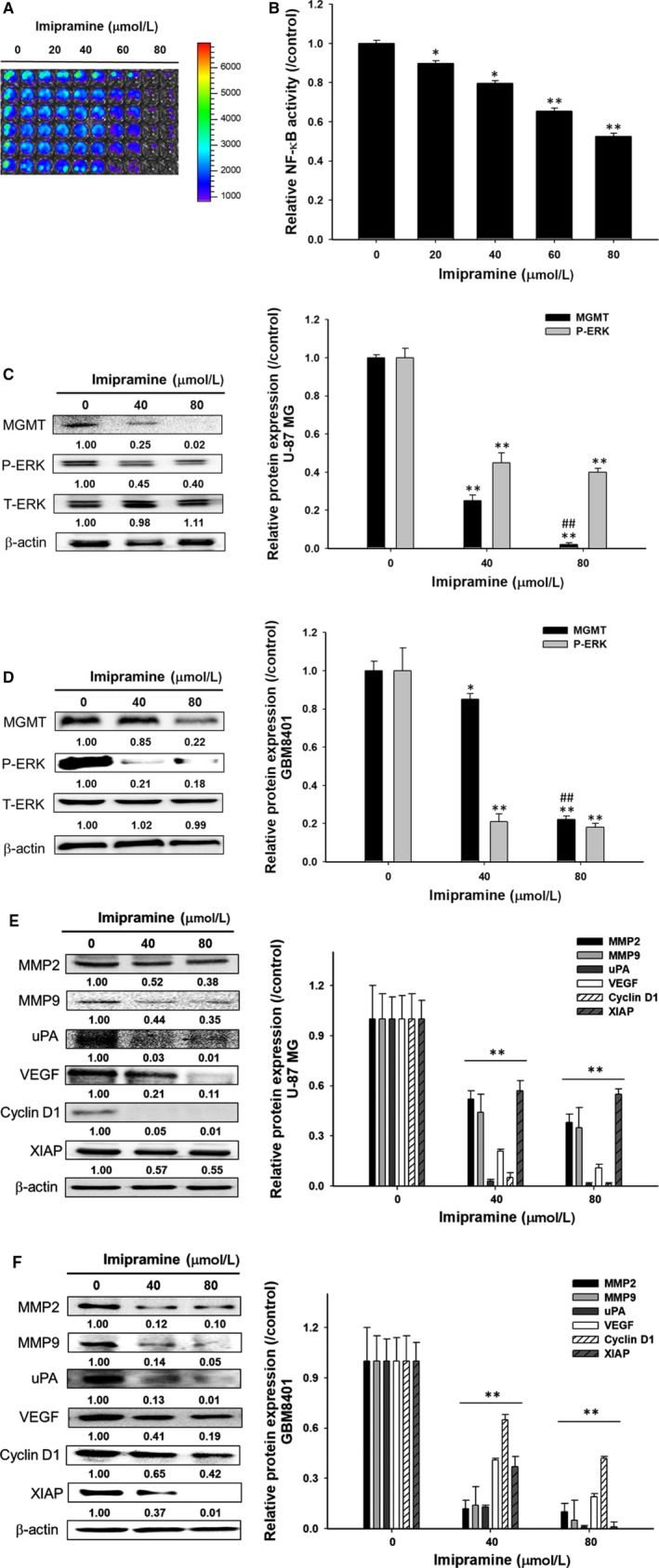
ERK/NF‐κB activity and associated tumour progression proteins were diminished by imipramine in vitro. U87/*NF‐κB*/*luc2* cells were treated with 0‐80 µmol/L imipramine for 48 h, and an NF‐κB reporter gene assay was performed. A, BLI colour‐coded images under various treatment doses are displayed. B, The relative NF‐κB activity was calculated and displayed as a bar chart. Expressions of the proteins MGMT, P‐ERK and T‐ERK after 0, 40 and 80 µmol/L of imipramine treatment on (C) U‐87 MG and (D) GBM8401 cells. Protein expressions of NF‐κB–related genes on (E) U‐87 MG and (F) GBM8401 cells. Relative protein quantification results of three repeated experiments are also displayed. **P < .01: significant difference between imipramine‐treated groups and the control as analysed by Student's *t* tests

In order to examine the mechanism by which imipramine affects the DNA damage/repair, proliferation, migration and invasion of glioblastoma cells, its effects on the levels of NF‐κB activation and certain proteins were examined. As shown in the reporter gene assay, imipramine markedly suppressed NF‐κB activation in U87*/NF‐*κ*B/luc2* cells (Figure 6A). Imipramine was found to significantly reduce the NF‐κB upstream mediator protein phosphorylation level of p‐ERK in both types of glioblastoma cells (Figure 6C,D). In addition, the DNA damage repair‐related marker MGMT was also effectively suppressed by imipramine (Figure 6C,D). Moreover, tumour progression‐related proteins such as MMP‐2, MMP‐9, uPA, VEGF, Cyclin D1 and XIAP were all decreased by imipramine in U‐87 MG and GBM8401 cells (Figure 6E,F). Based on these observations, it is concluded that imipramine inhibits tumour progression through the inhibition of the ERK//NF‐κB pathway.

### 3.7 Imipramine suppressed the growth of U‐87 MG tumour xenograft in vivo

**Figure 7:**
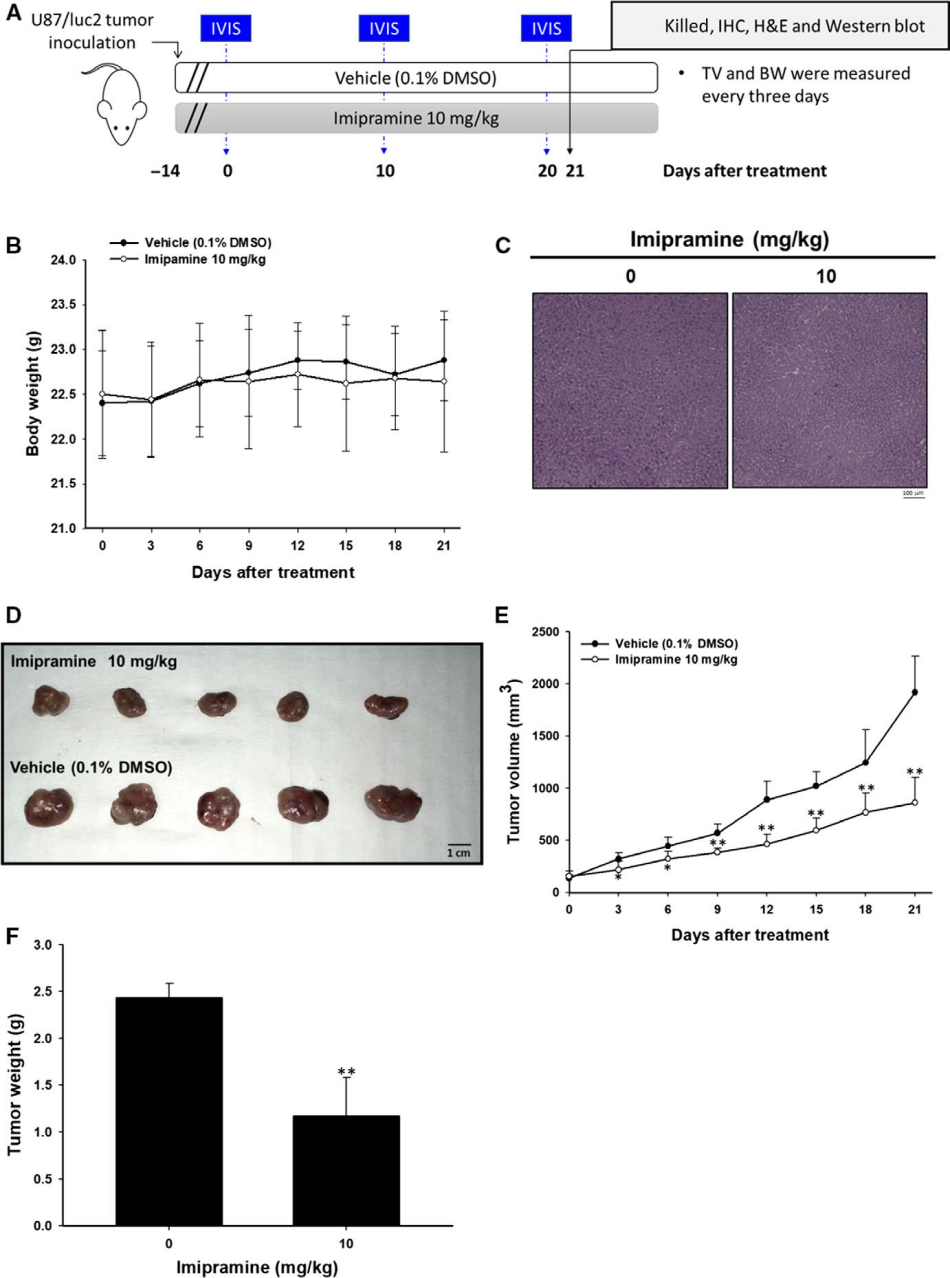
No general toxicity, but tumour growth inhibition, was found in imipramine‐treated glioblastoma‐bearing mice. A, Animal experimental procedure. B, Mice body weight was recorded every 3 days and calculated. C, Mice liver tissue was investigated with H&E staining. D, Tumour tissues extracted from mice on day 21 are displayed. E, Tumour volume was also recorded every 3 days. F, Tumour weight was measured after extraction from mice. **P < .01: significant difference between imipramine‐treated groups and vehicle as analysed by Student's *t* test

An animal experimental flowchart is displayed in Figure 7A, and all experiments were repeated twice. A total of 10 mice was divided into 2 groups: a 0.1% DMSO‐treated vehicle group and a 10 mg/kg imipramine‐treated group. BLI scan was performed on days 0, 10 and 20 after treatment. Tumour volume (TV) and body weight (BW) were measured every three days. Mice were killed on day 21 for IHC, H&E staining and ex vivo Western blotting. In Figure 7B, the mice's body weight remained unchanged during 21 days of treatment. The lack of an obvious liver pathology difference between vehicle and imipramine group mice indicated that no general toxicity was found (Figure 7C). The tumours extracted on day 21 showed that sizes in the imipramine‐treated group were markedly smaller than the vehicle group (Figure 7D). In Figure 7E, effective tumour growth inhibition was found in imipramine group after 3‐day administration until the end of the experiment. Moreover, the tumour weight from both groups was measured after being isolated from killed mice. As can be seen in Figure 7F, the tumour weight was effectively reduced by imipramine, and this result corresponded with Figure 7D,E.

### 3.8 Imipramine suppressed tumour growth via inactivation of ERK/NF‐κB signalling transduction

**Figure 8:**
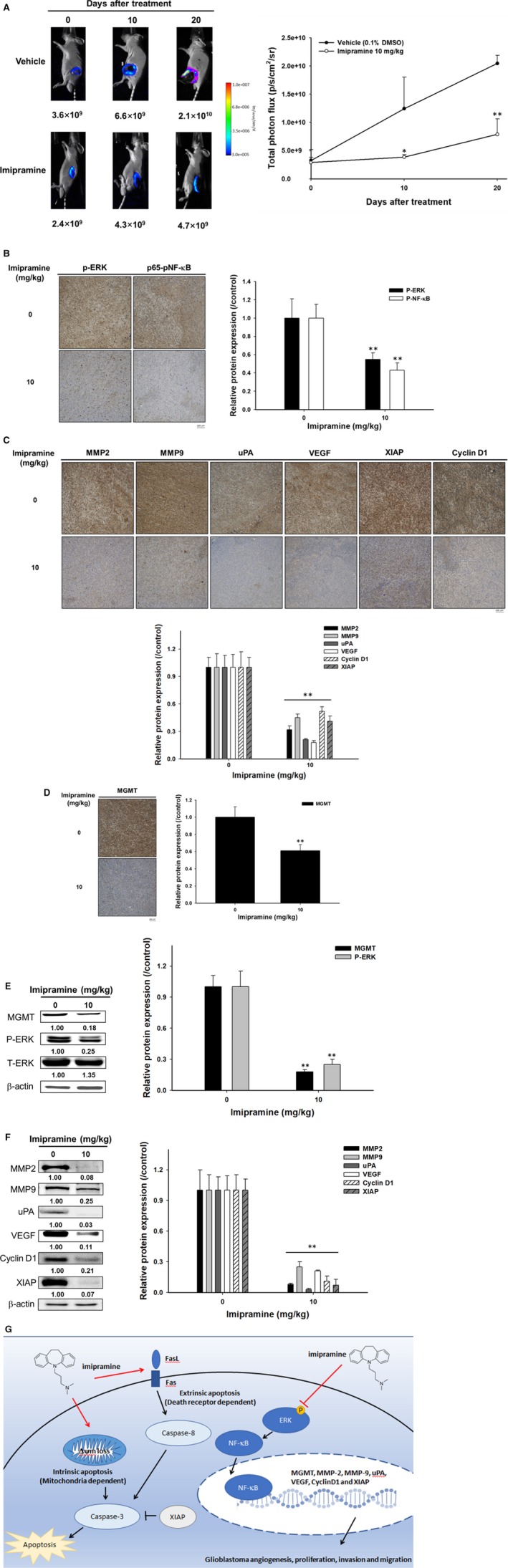
ERK/NF‐κB activity and associated tumour progression proteins were diminished by imipramine on glioblastoma‐bearing mice. A, Representative IVIS images from both groups on day 0, 10 and 20 are displayed. NF‐κB activation was calculated by drawing a relative region of interests (ROI) on Living Image software. B, Protein expressions of P‐ERK and P‐P65 NF‐κB (Ser 276) from mice tumours were measured by IHC staining. C, Protein expressions of NF‐κB–related genes from mice tumours were measured by IHC staining. D, Protein expressions of MGMT from mice tumours were measured by IHC staining. Ex vivo Western blot results of (E) MGMT, P‐ERK, P‐P65 NF‐κB– and (F) NF‐κB–related proteins of tumour tissue isolated from mice. G, Summarized mechanism of imipramine on glioblastoma. **P < .01: significant differences between imipramine‐treated groups and vehicles as analysed by Student's *t* tests

To identify whether the NF‐κB inhibition effect of imipramine also worked effectively on in vivo animal models, we performed in vivo NF‐κB activation analysis by IVIS. The result in Figure 8A showed the dramatic suppression of NF‐κB activation found in imipramine‐treated mice as compared to vehicle mice. The signal intensity of NF‐κB was markedly reduced in imipramine tumours. Then, we further validated whether NF‐κB upstream signalling was also suppressed by imipramine. In IHC staining results, the phosphorylation of ERK and phosphorylation of P‐65 NF‐κB were both inhibited by imipramine (Figure 8B). NF‐κB–associated proteins involved in tumour progression, such as MMP‐2, MMP‐9, uPA, VEGF, XIAP and Cyclin D1, were all decreased by imipramine (Figure 8C). Importantly, MGMT, which is involved in the treatment‐induced resistance of glioblastomas, was also decreased by imipramine (Figure 8D). Furthermore, ex vivo Western blotting was also performed to doubly confirm the protein expression alteration of imipramine treatment. As shown in Figure 8E,F, P‐ERK(Thr202/Tyr204), P‐P65 NF‐κB(Ser276), MGMT and NF‐κB–related protein expression was decreased 70%‐80% by imipramine as compared with vehicle treatment. The proposed mechanism of imipramine was displayed as Figure 8G.

## 4 DISCUSSION

Pro‐apoptotic proteins, the critical components of extrinsic and intrinsic apoptotic pathways, are decreased in glioblastomas.^41^, ^42^ Caspase family members act as activators, executioners or mediators modulating the induction, transduction and amplification of apoptotic signalling cascades. Caspase‐8, the apoptotic activator in the extrinsic pathway, can be activated with death receptor/death receptor‐ligand interaction. Caspase‐3, the apoptotic executioner, participates in apoptotic deoxyribonucleic acid (DNA) fragmentation.^43^ Decreased expression of active caspase‐8 and caspase‐3 were recognized as poor prognostic biomarkers related to worse survival in patients with glioblastomas.^42^, ^44^ Our data showed that imipramine significantly increased the activation of both caspase‐3 and caspase‐8 in glioblastoma U87 MG and GBM‐8401 cells (Figures 3 and 4). In addition, the down‐regulation of apoptotic pathways and up‐regulation of anti‐apoptotic proteins were associated with apoptosis evasion, contributing to tumour progression.^45^ The overexpression of anti‐apoptotic proteins abrogates chemotherapeutic agent‐induced cell death through the disruption of apoptotic signalling transduction.^46^ X‐linked inhibitor of apoptosis (XIAP), the anti‐apoptotic protein, hinders chemotherapy‐induced apoptosis by the reduction of caspase‐3, ‐7 and ‐9 activation.^47^ Glioblastoma patients with high XIAP expression had worse survival rates than those with low XIAP expression.^48^ As shown in Figures 6 and 8, XIAP expression was significantly decreased by imipramine in glioblastoma U87 MG and GBM‐8401 cells.

Loss of mitochondrial membrane potential (ΔΨ_m_) activates caspase‐mediated apoptosis through the release of cytochrome C and apoptotic protease activating factor 1 (APAF1) from mitochondria.^49^ Jeon et al^23^ demonstrated that imipramine triggered autophagic cell death (type II programmed cell death) in glioblastoma U‐87 MG cells. Imipramine also exhibited the ability to induce the loss of ΔΨ_m_ and decrease the expression of B‐cell lymphoma 2, (Bcl‐2).^50^ Figures 4 and 5 indicate that imipramine significantly induced apoptosis, extrinsic (increased activation of Fas, Fas‐L and caspase 8) and intrinsic (loss of ΔΨ_m_) apoptotic signalling. In addition, we found that imipramine may also increase the accumulation of Ca^2+^ (Figure 5C,D). Importantly, the accumulation of Ca^2+^ is associated with endoplasmic reticulum stress‐induced apoptosis.^51^

A high expression of tumour progression‐associated proteins, such as MMP‐9, MMP‐2, uPA, VEGF and Cyclin D1, was associated with poor outcomes in high‐grade glioma.^52^, ^53^, ^54^, ^55^, ^56^ MMP‐9, MMP‐2 and uPA promote tumour cell invasion through the digestion of extracellular matrix (ECM) components.^54^, ^57^ Angiogenesis mediated by VEGF is essential for tumour growth and metastasis.^58^ Cyclin D1 regulates cell cycle progression by the acceleration of G_1_/S transition.^57^ The down‐regulation of tumour progression‐associated proteins not only inhibited glioblastoma growth and invasion but also sensitized glioblastoma to TMZ.^56^, ^59^, ^60^, ^61^ Our transwell results showed that imipramine significantly reduced the invasion/migration ability (Figure 2E‐H) and expression of tumour progression‐associated proteins in glioblastoma (Figures 6E,F and 8). Furthermore, MGMT, the DNA repair protein, inhibits TMZ‐induced cytotoxicity by the removal of DNA damage. The induction of MGMT expression was related to TMZ insensitivity, and the decreased protein level of MGMT, as a favourable prognostic factor, was associated with better prognosis in patients with glioblastoma.^9^, ^62^ Yu et al^15^ indicated that the expression of MGMT was regulated by NF‐κB activation. Previous studies demonstrated that the blockage of NF‐κB activation by specific NF‐κB inhibitors suppressed the expression of tumour progression‐associated proteins (XIAP, MMP‐2, MMP‐9, VEGF and Cyclin D1) and reduced cell invasion in glioblastoma.^27^, ^29^ Here, we found that imipramine may effectively reduce the protein levels of MGMT and NF‐κB activation in glioblastoma (Figures 6A‐D and 8D).

Phosphor‐ERK (p‐ERK), the critical kinase activated by Raf/MEK signalling, up‐regulates downstream oncogenic kinases and transcription factor‐mediated tumour cell survival, growth and invasion, resulting in tumour progression.^63^ The expression of ERR was increased in glioblastoma, and ERK activation was associated with the pathology of glioblastoma. Lopez‐Gines et al^64^ suggested that PD98059 (the inhibitor of MEK/ERK signalling) diminished NF‐κB activation in glioblastoma. ERK has been recognized as an important therapeutic target for glioblastoma.^29^ Sorafenib, the oral multikinase inhibitor approved for the treatment of hepatocellular carcinoma, modulates ERK dephosphorylation by targeting Raf.^65^, ^66^ Sorafenib‐inhibited NF‐κB activation was associated with ERK dephosphorylation,^67^ whereas limited activity was found in the combination of sorafenib and TMZ in patients with relapsed glioblastoma.^25^, ^68^ In our Western and IHC data, we provided evidence that imipramine significantly restrained the protein level of p‐ERK in glioblastoma (Figures 6C,D and 8B,E).

Here, we summarized our proposed mechanism of imipramine in glioblastoma (Figure 8G). First, imipramine may inhibit ERK/NF‐κB signalling transduction and thus reduced tumour progression‐related proteins expression, including MGMT, MMP‐2, MMP‐9, uPA, VEGF, Cyclin D1 and XIAP. Thus, tumour progression of glioblastoma was effectively blocked by imipramine treatment. In addition, imipramine may also trigger death receptor‐dependent extrinsic and mitochondria‐dependent intrinsic apoptosis signalling. In sum, the inhibition of ERK/NF‐κB signalling transduction and the induction of apoptosis are associated with imipramine‐inhibited tumour progression. In conclusion, we suggest that imipramine may be used as a potential complementary agent, affording additional therapeutic efficacy to patients with glioblastomas. Thus, validated the progression‐free survival data from patients who taking this antidepressant may be our further step.

## CONFLICTS OF INTEREST

The authors declare no conflicts of interest.

## AUTHORS' CONTRIBUTION

Hsu F.‐T. contributed to data curation; Hsu F.‐T., Chiang I.‐T. and Wang W.‐S. contributed to funding acquisition, writing‐original draft and writing‐review and editing.

## Data Availability

All data supporting the findings of this study are available within the article, within its supplementary informationfiles and from the corresponding author upon reasonable request.
