# Supplementary material for: Induction of apoptosis through extrinsic/intrinsic pathways and suppression of ERK/NF‐κB signalling participate in anti‐glioblastoma of imipramine
Source: J Cell Mol Med. 2020 Mar 9;24(7):3982–4000. doi: 10.1111/jcmm.15022 (PMC7171418; doi:10.1111/jcmm.15022)

Induction of apoptosis through extrinsic/intrinsic pathways and suppression of ERK/NF-κB signaling participate in anti-glioblastoma of imipramine

Supplementary materials: Western blot whole blot images of figures 6 and 8.

Figure 6C. (Right panel indicated the display images in manuscript.)

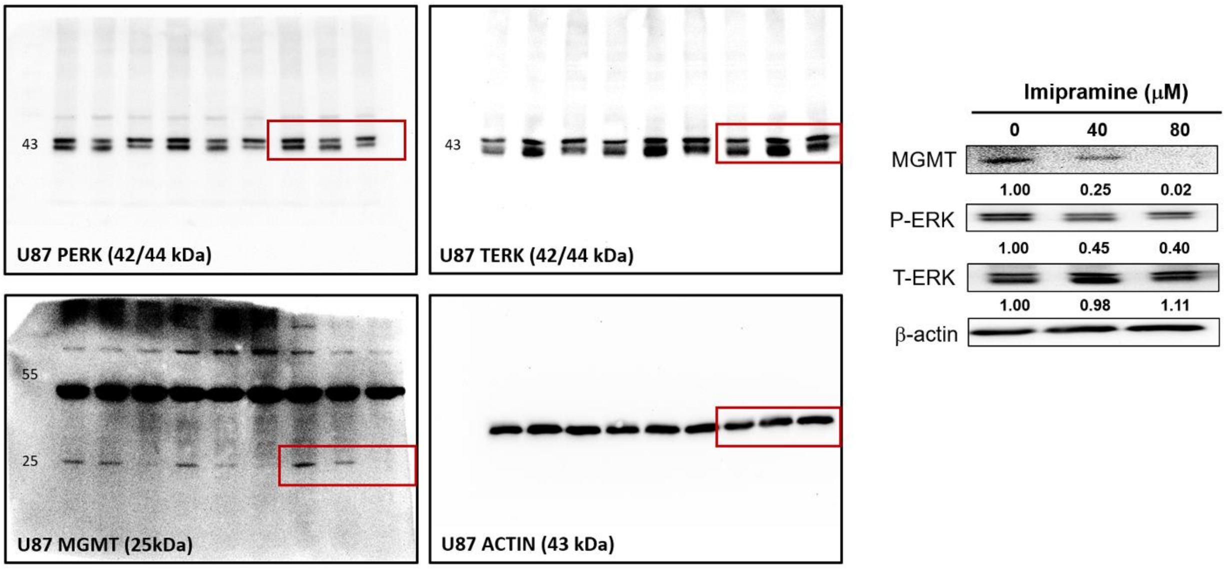

Figure 6D. (Right panel indicated the display images in manuscript.)

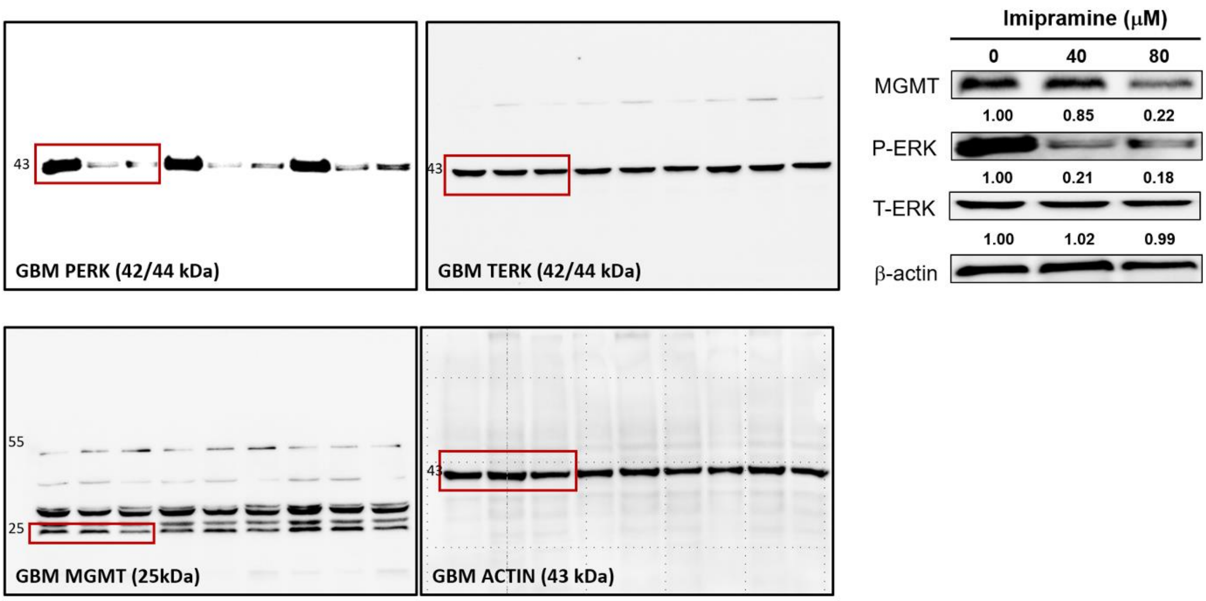

Figure 6E. (Right panel indicated the display images in manuscript.)

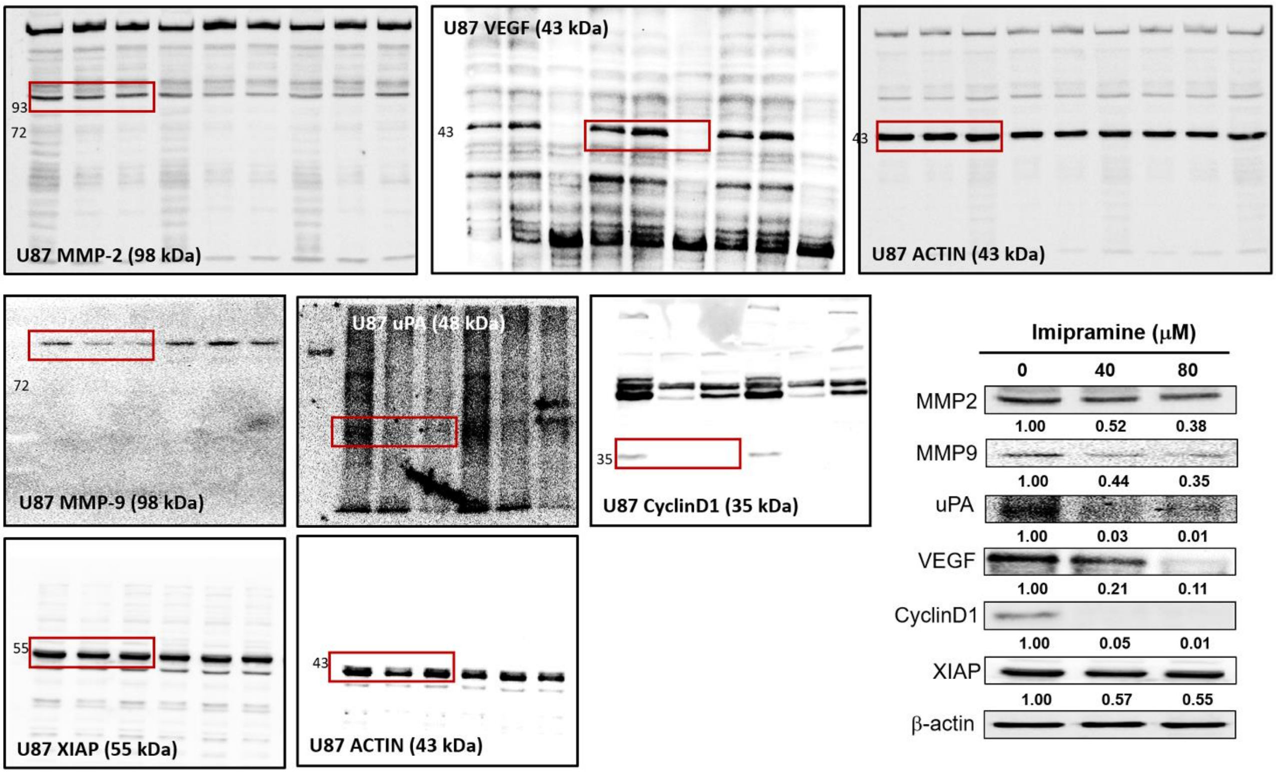

Figure 6F. (Right panel indicated the display images in manuscript.)

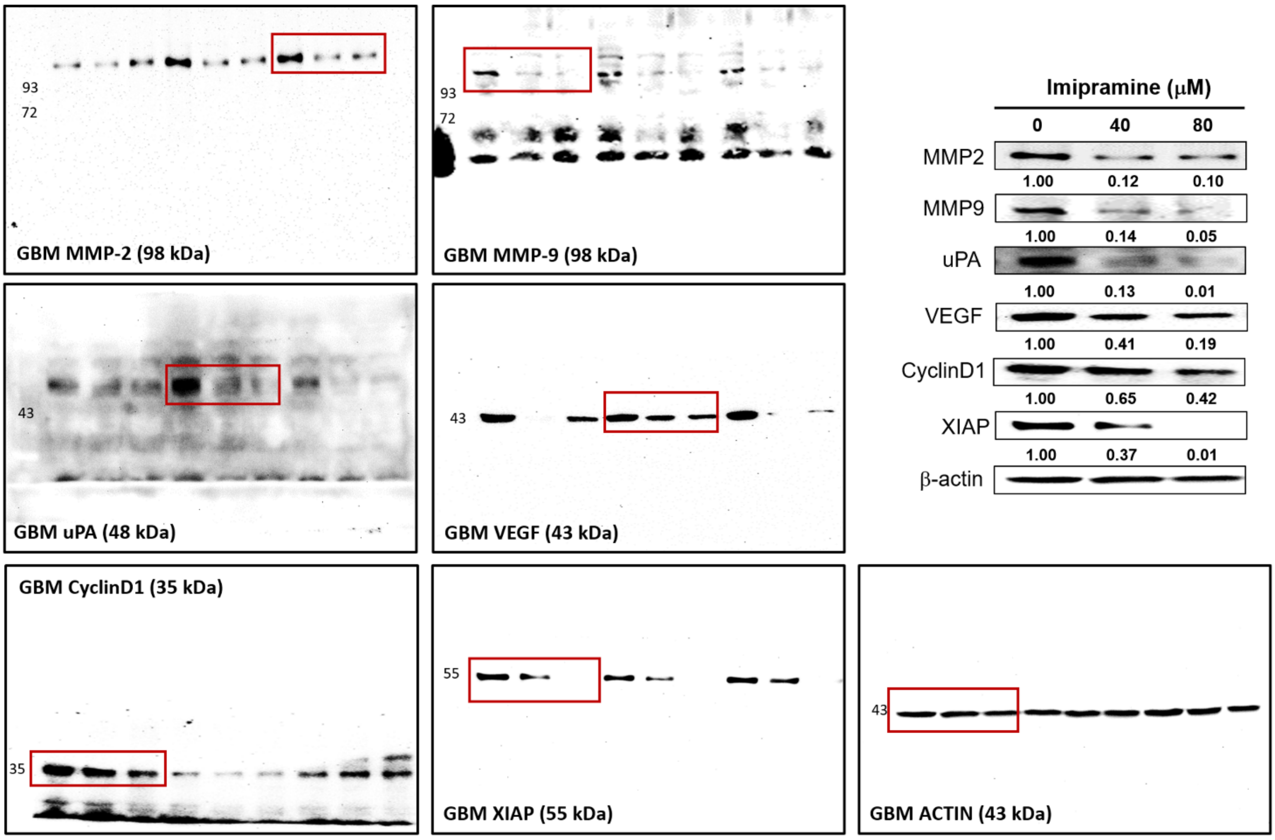

Figure 8E (Left panel indicated the display images in manuscript.)

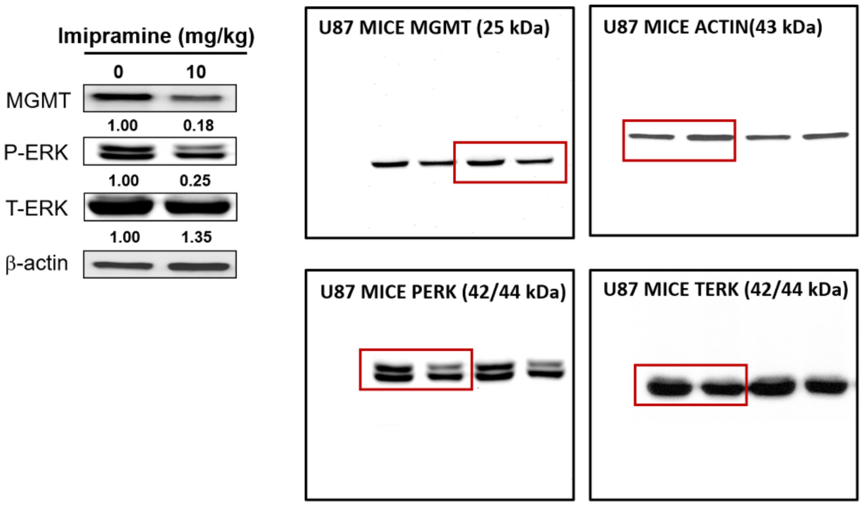

Figure 8F (Left panel indicated the display images in manuscript.)

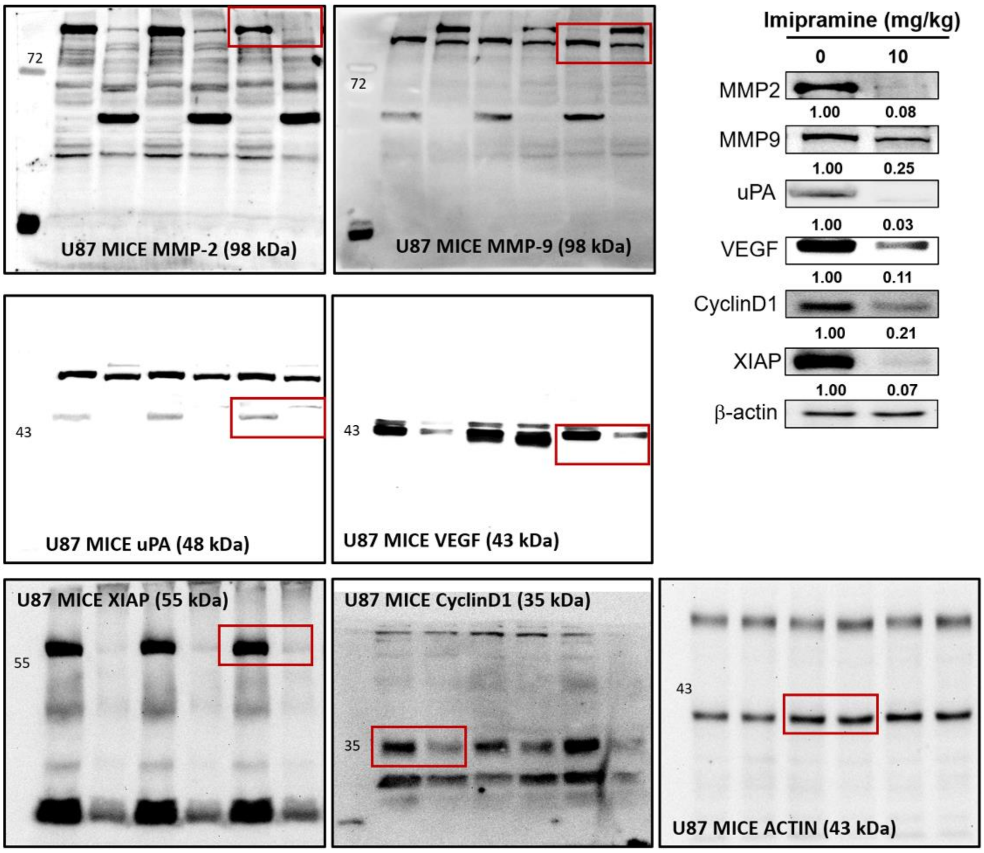

Supplement: Supplementary file 1 [file JCMM-24-3982-s001.pdf]
